# Family functioning and nicotine dependence among smoking fathers: a cross-sectional study

**DOI:** 10.1186/s12889-023-15475-4

**Published:** 2023-04-06

**Authors:** Yi Guo, Di-yue Liu, Yu-jia Wang, Meng-jie Huang, Nan Jiang, Qiang Hou, Bojunhao Feng, Wen-yu Wu, Yi-bo Wu, Fei Qi, Xin-ying Sun

**Affiliations:** 1 School of Public Health, Peking University, Beijing, China; 2 International School of Public Health and One Health, Hainan Medical University, Haikou, China; 3 College of Humanities and Social Sciences, Harbin Medical University, Heilongjiang, China; 4 School of Public Health, Shandong University, Jinan, China; 5 School of Public Health, Qingdao University, Qingdao, China; 6 College of Medical Sciences, Shanxi Medical University, Shanxi, China; 7 School of Medicine, Macau University of Science and Technology, Macao, China; 8 School of Health Management, Harbin Medical University, Heilongjiang, China; 9 Qingdao Municipal Center for Disease Control and Prevention, Qingdao, China

**Keywords:** Nicotine dependence, Family Functioning, Smoking fathers, Mcmaster model, Smoking cessation

## Abstract

**Background:**

Nicotine dependence is a significant public health issue, and understanding the factors associated with nicotine dependence in this population is crucial for developing effective interventions. This study examined the association between family functioning and nicotine dependence levels of smoking fathers based on the McMaster model of family functioning (MMFF), providing evidence for future interventions.

**Methods:**

In this study, we selected fathers of first- to fifth-grade students from 10 pilot elementary schools in Qingdao whose families smoked. We used the Fagerstrom test to assess nicotine dependence and the Family Assessment Device to evaluate family functioning. We performed univariate analysis to compare differences among those with different levels of nicotine dependence, and we used an ordinal logistic regression analysis to investigate the influences related to nicotine dependence.

**Results:**

This study included 874 smokers, with 78.5% having mild nicotine dependence, 11.7% having moderate dependence, and 9.84% having severe dependence. Univariate analysis showed that smokers with severe dependence had lower education levels, higher prevalence of chronic diseases, more frequent alcohol consumption, and poorer family functioning compared to those with mild to moderate dependence. Ordinal logistic regression analysis showed that poorer general functioning scores (OR = 1.087, 95% CI: 1.008–1.173, *P* = 0.030), poorer behavioral control (OR = 1.124, 95% CI: 1.026–1.232, *P* = 0.012), more quit attempts, frequent alcohol consumption, and longer smoking duration may be associated with a higher likelihood of developing severe nicotine dependence. The older age of starting smoking and higher education level may be associated with a lower likelihood of developing severe nicotine dependence. However, it is important to note that the cross-sectional nature of this study precludes the determination of causal relationships.

**Conclusions:**

This study finds that heavy nicotine dependence in smoking fathers is associated with risky behaviors and demographics such as longer smoking duration and frequent alcohol consumption. Targeted smoking cessation interventions are crucial for this group, taking these specific factors into consideration. Family functioning, particularly general functioning and behavioral control, may also be linked to nicotine dependence, indicating the need for further research in this area.

**Supplementary Information:**

The online version contains supplementary material available at 10.1186/s12889-023-15475-4.

## Introduction

It is an established fact that tobacco smoking has deleterious effects on human health. The World Health Organization’s (WHO) 2019 report reveals that smoking accounts for over 8 million deaths worldwide annually, with 7 million of these deaths being due to smoking-related diseases and 1.2 million due to secondhand smoke exposure. Despite the well-known benefits of smoking cessation in reducing the risk of smoking-related diseases [1, 2], it remains challenging for smokers to quit smoking due to nicotine dependence. Like other addictive disorders, nicotine dependence is characterized by physical and psychological withdrawal symptoms [3]. In China, a low proportion of smokers attempt to quit smoking, and awareness of quitting is poor, [4]. Many studies have demonstrated nicotine dependence is a most consistent and significant variable affecting quit attempts [5–7]. Therefore, understanding the level of tobacco nicotine dependence among smokers is critical for effective cessation.

Smoking cessation interventions often take place in a variety of settings, such as the home [8], the community [9], occupational settings [10], and hospitals [11]. But regardless of the environment smokers are in, their smoking cessation management includes the family and can be influenced by family functioning. [12, 13]. Family is the basic unit of society and an important site for the physical and psychological development of the individual. Given the vital role of the family at the individual level, over the past 20 years, a growing number of researchers have moved away from past research paradigms that explored one or some family factors in isolation and toward examining the family as a system. As a result, family functioning, which better reflects the family as a system, has received attention from researchers and has increasingly become a hot research topic. In recent smoking-related studies, researchers have concluded that smoking cessation cannot be studied only at the individual level and that smoking cessation interventions through the family have become a hot research topic. Research on the relationship between family and smoking cessation is evolving [14].

Despite the growing interest in family-based smoking cessation interventions, few studies have applied comprehensive family-related theories or measurement tools to understand the role of family functioning in nicotine dependence. It has been suggested that identifying the attributes of family functioning can provide guidelines for optimizing health-related interventions in clinical settings [15].

In the academic field, there are numerous definitions of family functioning, and several theories have been developed to understand it. Two of the most influential theories are the circumplex model theory and the McMaster model of family functioning. However, the circumplex model theory is mainly used to identify the problems in a family or evaluate the effectiveness of family treatment, and is primarily used in families with a member who has a mental health condition. Conversely, the McMaster model of family functioning focuses on the specific aspects of family functioning and their practical effects, avoiding the disadvantage of being too formalized. Due to its wider applicability to the general healthy population, this study employs the McMaster model to investigate family functioning.

The McMaster model of family functioning provides a comprehensive framework for understanding the functions of the family [16]. According to this model, the primary function of the family is to create a supportive and nurturing environment for the physical, psychological, and social development of its members. In order to fulfill this primary function, the family system must accomplish a range of tasks, such as meeting the basic material needs of individuals, adapting to and promoting the development of the family and its members, and managing various family crises. The family’s ability to fulfill its basic functions and tasks is mainly manifested in seven dimensions: general functioning, problem-solving, communication, role, affective responsiveness, emotional involvement, and behavioral control. Among them, problem-solving and communication refer to the family’s capacity to address and resolve problems effectively and communicate in a clear and constructive manner, respectively. Role pertains to the relative positions of family members, the responsibilities they assume, and the correspondingbehavioral expectations. Affective responsiveness concerns the ability to express and regulate emotions in response to specific stimuli. Emotional involvement refers to the degree of emotional closeness between family members, the family’s recognition and respect for each individual’s personality and preferences, and the extent to which individual needs are met. Finally, behavioral control relates to the family’s ability to regulate and manage the behavior of its members, including setting boundaries and tolerating differences.

Family is an significant environment where smoking occurs [17], and family support family support has shown to be a promising strategy for smoking cessation [18]. Hence, this study aims to investigate the relationship between family functioning and the level of nicotine dependence in smokers using the McMaster model of family as a theoretical framework. By examining the different domains of family functioning, this study seeks to shed light on the potential impact of family dynamics on nicotine dependence, which could inform the development of effective interventions to help smokers quit smoking.

## Methods

### Data source and sampling procedure

This study was conducted in 11 pilot primary schools located in Qingdao, involving smoking fathers with children in the first to fifth grades.(As the experiment was conducted after the sixth-grade students had graduated, it was not possible to contact their parents through the school. Therefore, only students from grades one to five were included in the study.) The initial screening of potential participants who met the inclusion criteria was carried out by investigators, followed by telephone and face-to-face interviews to obtain further information about the smoking fathers. Fathers who met the inclusion criteria and had no exclusion criteria matters were asked if they were willing to participate in the study. Those who declined to participate were excluded. The final list of respondents was formed by class, and the investigator obtained informed consent from the participants, providing them with detailed information about the study’s purpose, content, mode, benefits and risks of participating, and their rights and interests as participants. Participants were included in the research and invited to complete an online questionnaire after fully understanding the relevant content.

Inclusion criteria included: (a) fathers, mothers, and children living together in the past 30 days; (b) fathers smoking one or more cigarettes per day in the past 30 days; (c) participants familiar with smartphone functions; and (d) participants signing an informed consent form and voluntarily participating in the study.

Exclusion criteria included: (a) severe heart, brain, lung, or hematologic disorders; (b) history of mental illness or other medical conditions that prevent understanding and answering questions; and (c) prior participation in other smoking cessation intervention programs.

### Measurement of variables

#### Fagerstrom test for nicotine dependence

nicotine dependence was assessed using the Fagerstrom test for nicotine dependence (FTND), which has been widely validated by scholars at home and abroad [19]. The scale consists of six questions: How soon do you smoke your first cigarette after waking up? Do you find it difficult not smoke in places where you shouldn’t? Which cigarette would you hate to give up; which cigarette do you treasure the most? How many cigarettes do you smoke each day? Do you smoke more during the first few hours after waking up than during the rest of the day? Do you still smoke if you are so sick that you are in bed most of the day, have a cold or flu, and have trouble breathing? The answers to each question were given a different score, up to a maximum of 10, with the higher the combined score, the higher the level of nicotine dependence.

#### Family assessment device

The scale is a family functioning instrument developed by Epstein et al. using the McMaster model of family as a theoretical guide [16] for people older than 12 years of age. It can quickly and effectively identify problems in the family. There are 60 items with 7 subscales: Problem-solving (PS), Communication (CM), Roles (RL), Affective Responsiveness (AR), Affective Involvement (AI), Behavior Control (BC), and General Functioning (GF). The number of items for each dimension is (AI)7, (AR) 6, (BC) 9, (CM) 9, (GF) 12, (PS) 6, and (RL) 11. Each item on the scale was rated on a 4-point Likert scale: “very much like my home”, “like my home”, “not like my home”, and “not at all like my home”. The scores were 1, 2, 3, and 4, respectively. The total score is 240. A higher score indicates worse family function; 60 to 120 is good, 121 to 180 is general, and 181 to 240 is poor. The Cronbach’s alpha coefficient of FAD is 0.860. It is widely used in survey research because of its good reliability and validity. [20]. Li Rongfeng et al. tested the revised Chinese version of the scale and confirmed its good reliability and validity [21].

#### Basic information questionnaire

The scale was self-designed and included age, education level, whether or not they drank alcohol, information about diseases, when they first smoked, whether or not they had ever quit, and how long they had smoked.

#### Indicator definition

nicotine dependence: The FTND score ranges from 0 to 10, with higher scores indicating greater nicotine dependence. In this study, nicotine dependence is defined as follows: 0–3 points on the FTND scale are considered mild nicotine dependence, 4–5 points are considered moderate nicotine dependence, and scores of 6 or higher are considered severe nicotine dependence.

Diseases: In this study, “disease” refers to any chronic medical condition that has been diagnosed by a physician and requires ongoing management, such as diabetes, hypertension, cardiovascular disease, autoimmune disorders, and others.

### Data analysis

Statistical analysis was performed using STATA 15.1 software. Descriptive statistics were used to describe the demographic characteristics and smoking-related variables of the participants. Continuous variables were reported as means and standard deviations (SDs). Categorical variables were presented as frequencies and percentages. The Chi-square test was used to compare categorical variables such as location and alcohol consumption. The Kruskal-Wallis test was used to compare the differences in family functioning scores, age and smoking duration among smokers.

Ordinal logistic regression analysis was performed to assess the associations between family functioning and nicotine dependence. The dependent variable was nicotine dependence, as measured by the Fagerstrom test for nicotine dependence (FTND), and the independent variable was family functioning, as measured by the Family Assessment Device (FAD). Potential confounding variables, such as age, alcohol consumption, disease history, and age of starting smoking, were included in the multivariate logistic regression model. Odds ratios (ORs) and 95% confidence intervals (CIs) were calculated to estimate the strength of the associations. A p-value < 0.05 was considered statistically significant. Subgroup analysis was performed by education level, and the results were reported separately.

### Ethical consideration

This study has been reviewed and approved by the Medical Ethics Committee of the Qingdao Center for Disease Control and Prevention, with project number 2021-ZXJK-32.

## Results

Out of the 874 smokers who participated in this study, 686 (78.5%) had mild nicotine dependence, while 102 (11.7%) had moderate dependence, and 86 (9.84%) had severe nicotine dependence. The mean age of the participants was 39.85 (SD = 5.78) years, and the mean age of starting smoking was 27.77 (SD = 9.19) years. A majority of the participants, 715 (81.90%), reported starting smoking after the age of 18. The mean smoking duration was 12.06 (SD = 8.90) years.

Statistical analysis showed significant differences (P < 0.05) between groups with different levels of nicotine dependence in terms of education level, prevalence of chronic diseases, frequency of alcohol consumption, age of starting smoking, and number of quit attempts. Compared with smokers with mild to moderate nicotine dependence, smokers with severe nicotine dependence had lower education levels, higher prevalence of chronic diseases, and more frequent alcohol consumption. In addition, concerning smoking-related characteristics, severe tobacco-dependent smokers were more likely to have started smoking before 18, to have smoked for longer, and to have experienced more smoking cessation. In this study, no significant association was found between the type of occupation and the degree of nicotine dependence. (Table 1)

**Table 1:** Statistical description of demographic characteristics and smoking-related characteristics and degree of nicotine dependence [Number (%)]

Grouping variables	Overall (N = 874)	Mild nicotine dependence	Mild nicotine dependence	Severe nicotine dependence	*χ* ^*2*^ */H*
**Age**	39.85 ± 5.78	39.90 ± 5.58	39.37 ± 6.14	39.94 ± 6.88	1.28
**Smoking duration**	12.06 ± 8.90	10.75 ± 8.60	15.68 ± 8.67	18.06 ± 7.95	66.61^**^
**Education level**					21.00^**^
High school and below	348(39.82%)	246(70.69%)	54(15.52%)	48(13.79%)	
College and above	526(60.18%)	440(83.65%)	48(9.13%)	38(7.22%)	
**Chronic diseases**					10.22^*^
No disease	777(88.90%)	620(79.79%)	89(11.45%)	68(8.75%)	
Illness	97(11.10%)	66(68.04%)	13(13.40%)	18(18.56%)	
**Alcohol consumption**					34.42^**^
No drinking	295(28.03%)	247(83.73%)	26(8.81%)	22(7.46%)	
Drinking less than 1 time per month	119(13.62%)	98(82.35%)	11(9.24%)	10(8.40%)	
Drinking 1–3 days a month	245(28.03%)	197(80.41%)	29(11.84%)	19 (7.76%)	
Drinking 1–3 days per week	143(16.36%)	103(72.03%)	23(16.08%)	17(11.89%)	
Drinking 4–7 days per week	72(8.24%)	41(56.94%)	13(18.06%)	18(25.00%)	
**Job**					2.26
White-collar	659(75.40%)	521(79.06%)	73(11.08%)	65(9.86%)	
Blue-collar	178(20.37%)	138(77.53%)	22(12.36%)	18(10.11%)	
Other	37(4.23%)	27(72.97%)	7(18.92%)	3(8.11%)	
**Age of first smoking**					53.61^**^
< 18	158(18.08%)	92(58.23%)	29(18.35%)	37(23.42%)	
≥ 18	716(81.92%)	594(82.96%)	73(10.20%)	49(6.84%)	
**Quit attempts**					20.64^**^
Less than twice	757(86.61%)	612(80.95%)	82(10.85%)	63(8.20%)	
Twice of more	117(13.39%)	74(63.25%)	20(17.09%)	23(19.66%)	

^**^P < 0.001,^*^P < 0.05;

Based on the Family Functioning Scale completed by the participants in this study, it was found that the participants generally scored lower in General Functioning (GF) but higher in Affective Responsiveness (AR). The overall total score of the scale was (125.35 ± 20.13), with the highest mean score for General Functioning (GF) being (24.14 ± 5.32), and the lowest mean score for Affective Responsiveness (AR) being (12.77 ± 2.47).

The study found a significant difference in family functioning scores across the three groups of smokers with varying levels of nicotine dependence, as determined by the Kruskal-Wallis test. Specifically, smokers with higher levels of nicotine dependence had poorer family functioning compared to those with lower levels of dependence. The results showed statistically significant differences (P < 0.05) in mean scores for several dimensions of family functioning, including Communication, Roles, Affective Responsiveness, Affective Involvement, Behavior Control, and General Functioning. These findings suggest that heavy nicotine dependence may be associated with higher scores on the family functioning scale, indicating poorer overall family functioning.(Table 2).

**Table 2:** Statistical description of FAD scores and the degree of nicotine dependence of the participants ($\overline{x}$±s, points)

Continuous Variables	Overall (N = 874)	Mild nicotine dependence	Mild nicotine dependence	Severe nicotine dependence	*H*
Total FAD score	125.35 ± 20.13	124.78 ± 21 0.43	133.06 ± 20.12	134.71 ± 17.61	25.87^**^
PS	13.28 ± 4.39	13.07 ± 4.61	13.19 ± 3.79	13.45 ± 3.89	2.12
CM	19.24 ± 3.93	19.11 ± 4.06	20.03 ± 2.95	20.41 ± 3.45	10.60^*^
RL	22.71 ± 4.12	22.71 ± 4.57	24.60 ± 4.10	24.41 ± 4.01	27.36^**^
AR	12.77 ± 2.47	12.74 ± 2.57	13.48 ± 2.32	13.77 ± 2.55	15.90^**^
AI	14.02 ± 3.84	14.09 ± 4.37	15.62 ± 4.26	15.40 ± 3.91	22.75^**^
BC	19.20 ± 3.04	19.12 ± 3.25	20.50 ± 22.93	20.79 ± 2.89	42.50^**^
GF	24.14 ± 5.32	23.94 ± 5.52	25.65 ± 4.41	26.49 ± 4.75	19.53^**^

^**^P < 0.001,^*^P < 0.05

In this study, ordinal logistic regression analysis was utilized to identify factors associated with heavy nicotine dependence. The analysis revealed that poor performance in general functioning(OR = 1.087, 95%CI: 1.008 ~ 1.173, *P* = 0.030) and behavioral control(OR = 1.124 95%CI: 1.026 ~ 1.232, *P* = 0.012), having made two or more quit attempts(OR = 1.790, 95% CI: 1.134 ~ 2.825, *P =* 0.012), more frequent alcohol consumption(OR = 1.220, 95% CI: 1.062 ~ 1.402, *P =* 0.005), and longer smoking duration(OR = 1.065, 95% CI: 1.033 ~ 1.098, *P* < 0.001) were all associated with severe nicotine dependence. Older age of starting smoking(OR = 0.930, 95% CI: 0.903 ~ 0.949, *P <* 0.001) and higher education level(OR = 0.650 95% CI: 0.442 ~ 0.950, *P* = 0.027) were associated with lower level of nicotine dependence.(Table 3)

**Table 3:** Ordered logistic regression analysis affecting the level of tobacco dependence

	*β*	SE	*P*	OR	95% CI
**Age**	-0.04	0.019	0.061	0.960	0.928 ~ 1.002
**Alcohol consumption**	**0.24**	**0.086**	**0.005**	**1.220**	**1.062 ~ 1.402**
**Education level** (control group = Non–highly educated)	**-0.28**	**0.127**	**0.027**	**0.650**	**0.442 ~ 0.950**
**Chronic diseases** (control group = No disease)	0.62	0.397	0.122	1.504	0.897 ~ 2.522
**Age of starting smoking** (control group = < 18)	**-0.07**	**0.012**	**< 0.001**	**0.930**	**0.903 ~ 0.949**
**Quit attempts** (control group = Less than twice)	**1.04**	**0.417**	**0.012**	**1.790**	**1.134 ~ 2.825**
**Smoking duration**	**0.07**	**0.017**	**< 0.001**	**1.065**	**1.033 ~ 1.098**
**FAD(GF)**	**0.09**	**0.042**	**0.030**	**1.087**	**1.008 ~ 1.173**
**FAD(PS)**	-0.03	0.044	0.550	0.973	0.891 ~ 1.063
**FAD(CM)**	0.01	0.050	0.891	1.007	0.914 ~ 1.109
**FAD(RL)**	-0.06	0.041	0.161	0.941	0.865 ~ 1.024
**FAD(AR)**	-0.04	0.056	0.431	0.955	0.850 ~ 1.072
**FAD(AI)**	0.00	0.052	0.943	0.997	0.911 ~ 1.091
**FAD(BC)**	**0.13**	**0.053**	**0.012**	**1.124**	**1.026 ~ 1.232**
**Job** (control group = White-collar)	Ref.	-	-	-	-
**Blue-collar**	-0.18	0.188	0.326	0.792	0.498 ~ 1.261
**Other**	-0.01	0.421	0.978	0.988	0.429 ~ 2.276
**School** (control group = School 1)	Ref.	-	-	-	-
**School = 2**	0.67	0.717	0.353	1.541	0.619 ~ 3.833
**School = 3**	1.05	1.403	0.452	1.798	0.390 ~ 8.295
**School = 4**	1.13	0.956	0.240	1.841	0.665 ~ 5.093
**School = 5**	0.00	0.001	0.974	0.000	0.000 ~ 0.000
**School = 6**	-0.35	0.312	0.258	0.476	0.131 ~ 1.723
**School = 7**	-0.31	0.286	0.287	0.603	0.238 ~ 1.528
**School = 8**	-0.04	0.444	0.930	0.960	0.388 ~ 2.377
**School = 9**	0.72	0.713	0.313	1.578	0.651 ~ 3.824
**School = 10**	0.44	0.542	0.410	1.382	0.640 ~ 2.983
**School = 11**	0.85	0.695	0.223	1.664	0734 ~ 3.771

The reference category: mild tobacco dependence

^***^P < 0.001, ^*^P < 0.05

## Discussion

The findings of this study suggest that smoking fathers of elementary school students in Qingdao exhibit a lower prevalence of heavy nicotine dependence, with a corresponding FTND scale score of (2.11 ± 2.22) and heavy nicotine dependence rate of 9.84%, as compared to the national level. [22] These results may suggest the effectiveness of tobacco control measures implemented in Qingdao in recent years, including the establishment of a tobacco-free city. Furthermore, the increased awareness of the health consequences of smoking on family members, attributable to the presence of children, may have also contributed to the lower rate of heavy nicotine dependence observed among smoking fathers [23].

Moreover, the multifactorial analysis findings suggest that more frequent alcohol consumption, longer smoking duration, and more smoking cessation attempts are associated with severe nicotine dependence, while starting smoking at an older age and having higher education level were associated with lower level of nicotine dependence. Related studies [24, 25] have demonstrated that longer smoking duration, younger age of smoking initiation, and particularly starting smoking before the age of 18 are linked to a higher likelihood of severe nicotine dependence among current adult smokers, which is consistent with the findings of this study. This may be due to the immaturity of brain mechanisms prone to nicotine dependence at a younger age [26].In addition, as the duration of smoking increases, nicotine in tobacco is involved in strengthening brain circuits with the help of high-affinity isoforms of α4 and β2 subunits (α4β2 * nAChRs), promoting psychological and physiological dependence on tobacco [27]. These findings underscore the importance of targeted smoking cessation interventions that consider the specific factors associated with nicotine dependence among smoking fathers.

In addition, the present study found a correlation between the number of quitting times and the degree of nicotine dependence, with those who quit ≥ 2 times having a higher risk of severe nicotine dependence compared to those who quit < 2 times, which is similar to the findings of Yanting Zhao[4]. This may be related to the fact that those who failed to quit smoking developed psychological anxiety and became more dependent on tobacco. [28]Additionally, greater nicotine addiction may have made it more difficult to quit in the past, leading to an increase in failed attempts. However, the study by Zhao Jie et al. [29] showed that those who tried to quit were more likely to stop dependent on tobacco. Further research is necessary to fully comprehend the correlation between smoking cessation and nicotine dependence.

The study indicates a potential association between family functioning and nicotine dependence among smoking fathers, as those with lower scores in General Functioning and behavioral control exhibited higher levels of nicotine dependence. The negative association between family functioning and nicotine dependence may have several explanations. One possibility is that poor family functioning contributes to increased stress and emotional distress, which can in turn increase the risk of substance abuse. Another explanation is that nicotine dependence may disrupt family relationships and lead to social isolation, which can further exacerbate the negative effects of substance abuse on family dynamics. According to the analysis of the McMaster model of family, in families with good General Functioning, smoking fathers may be more likely to reduce or quit smoking out of concern for the health of their family members or on the advice of their family members; in families with good behavioral control, effective behavioral discipline and reward and punishment mechanisms among family members may also have a positive effect on smokers’ reduction. Despite few studies analyzing nicotine dependence in the context of family functioning theory, addictive behaviors share similarities. Prior research analyzing family functioning’s correlation with other addictive behaviors has obtained similar findings to this study. Adolescents’ internet addiction [30], drug dependence [31], and drug addiction [32] are associated with poorer family functioning. Furthermore, the family’s overall functioning as a life unit and family members’ education levels impact individual tobacco and alcohol use. [33]. Tobacco use behavior in the family often also affects the tobacco use behavior of children, and some studies suggest that many minors’ tobacco use habits originate in the family [34, 35].

This study provides valuable insights into the factors that contribute to nicotine dependence among smoking fathers of elementary school students in Qingdao, China. The findings highlight the importance of family functioning and behavioral control as potential factors that can affect the level of nicotine dependence. The study’s results also suggest the need for targeted smoking cessation interventions that take into account the specific factors associated with heavy nicotine dependence among smoking fathers. These interventions should focus on improving family functioning and providing health education to family members to reduce tobacco use in the home environment. The results of this study could inform the development of effective smoking cessation programs that can help reduce tobacco use and improve public health outcomes in China and beyond.

There are some limitations of this study. The participants were limited to fathers in one city, which may limit the generalizability of the findings to other populations. Additionally, the data collected was based on self-reported information, and we didn’t take smoking fathers’ original family functioning into consideration, which may be subject to bias and may not accurately reflect participants’ actual smoking behaviors. The cross-sectional design of the study also limits the ability to draw conclusions about causality or the directionality of the observed associations.

## Conclusion

In conclusion, this study suggests that heavy nicotine dependence among smoking fathers is associated with certain demographic and behavioral factors, such as more frequent alcohol consumption and longer smoking duration. The findings highlight the importance of targeted smoking cessation interventions for this population, taking into account these specific factors. The study also suggests a potential link between family functioning and nicotine dependence, emphasizing the need for future research to explore this relationship further. Despite the limitations of the study, the results provide valuable insights into factors that may contribute to heavy nicotine dependence among smoking fathers and inform strategies for effective smoking cessation interventions.

## Electronic supplementary material

Below is the link to the electronic supplementary material.

**Subgroup Analysis**: S1 Ordered logistic regression analysis affecting the level of tobacco dependence(subgroups: College and above)

## Data Availability

The raw data is anonymous, there is no risk of identification, and access to the raw data is possible with the knowledge and authorization of the corresponding author.
