# Supplementary material for: Family functioning and nicotine dependence among smoking fathers: a cross-sectional study
Source: BMC Public Health. 2023 Apr 6;23:658. doi: 10.1186/s12889-023-15475-4 (PMC10080741; doi:10.1186/s12889-023-15475-4)
Supplement: Supplementary file 1 — Subgroup Analysis: S1 Ordered logistic regression analysis affecting the level of tobacco dependence(subgroups: College and above) [file 12889_2023_15475_MOESM1_ESM.docx]

## Subgroup Analysis

S1 Ordered logistic regression analysis affecting the level of tobacco dependence(subgroups: College and above)

|  | *β* | SE | *P* | OR | 95% CI |
| --- | --- | --- | --- | --- | --- |
| **Age** | -0.05 | 0.030 | .218 | .962 | 0.904~1.023 |
| **Alcohol consumption** | 0.15 | 0.147 | 0.017 | 1.308 | 1.308~1.049 |
| **Chronic diseases**  (control group= No disease) | 1.92 | 0.437 | 0.657 | 1.179 | 0.570~20437 |
| **Age of first smoking**  (control group=<18) | -0.15 | 0.312 | 0.580 | 0.808 | 0.379~1.720 |
| **Quit attempts**  (control group=Less than twice) | 0.62 | 0.831 | 0.011 | 2.404 | 11.220~4.735 |
| **FAD(GF)** | 0.05 | 0.068 | 0.049 | 1.126 | 1.001~1.267 |
| **FAD(PS)** | -0.10 | 0.068 | 0.420 | 1.054 | 0.928~1.197 |
| **FAD(CM)** | 0.04 | 0.074 | 0.956 | 1.004 | 0.870~1.159 |
| **FAD(RL)** | 0.05 | 0.06 | 0.012 | 0.835 | 0.726~0.961 |
| **FAD(AR)** | -0.11 | 0.083 | 0.858 | 0.985 | 0.835~1.162 |
| **FAD(AI)** | -0.11 | 0.080 | 0.030 | 1.161 | 1.015~1.327 |
| **FAD(BC)** | 0.24 | 0.075 | 0.617 | 1.037 | 0.900~1.194 |
| **Job**  (control group= White-collar) |  |  |  |  |  |
| **Blue-collar** | -0.21 | 0.336 | 0.732 | 0.877 | 0.414~1.859 |
| **Other** | -0.01 | 0.001 | 0.983 | 0.000 | 0.000~0.000 |
| **School**  (control group =School 1) |  |  |  |  |  |
| **School=2** | 2.73 | 0.562 | 0.858 | 0.894 | 0.260~3.068 |
| **School=3** | 0.00 | 0.609 | 0.631 | 0.627 | 0.093~4.216 |
| **School=4** | 2.31 | 1.715 | 0.300 | 2.224 | 0.491~10.083 |
| **School=5** | 0.00 | 0.000 | 0.987 | 0.000 | 0.000~0.000 |
| **School=6** | -0.21 | 0.240 | 0.134 | 0.294 | 0.059~1.460 |
| **School=7** | 0.91 | 0.19 | 0.058 | 0.291 | 0.081~1.043 |
| **School=8** | 2.29 | 0.304 | 0.254 | 0.499 | 0.151~1.648 |
| **School=9** | 6.09 | 0.42 | 0.447 | 0.572 | 0.135~2.414 |
| **School=10** | 3.03 | 0.495 | 0.792 | 0.859 | 0.278~2.657 |
| **School=11** | 8.15 | 0.419 | 0.534 | 0.683 | 0.206~2.272 |

The reference category: mild tobacco dependence

^***^ *P*<.001, ^*^ *P*<.05

S2 Ordered logistic regression analysis affecting the level of tobacco dependence(subgroups: High school and below)

|  | *β* | SE | *P* | OR | 95% CI |
| --- | --- | --- | --- | --- | --- |
| **Alcohol consumption** | 0.35 | 0.147 | 0.017 | 1.309 | 1.050~1.632 |
| **Age of first smoking**  (control group=<18) | -0.07 | 0.017 | <0.001 | 0.926 | 0.893~0.959 |
| **Quit attempts**  (control group=Less than twice) | 2.11 | 0.832 | 0.011 | 2.407 | 1.223~4.738 |
| **FAD(RL)** | -0.15 | 0.060 | 0.012 | 0.835 | 0.726~0.961 |
| **FAD(AI)** | 0.17 | 0.079 | 0.029 | 1.161 | 1.016~1.328 |

The reference category: mild tobacco dependence

^***^ *P*<.001, ^*^ *P*<.05
